# Fear, Stress, and Knowledge regarding COVID-19 in Nursing Students and Recent Graduates in Mexico

**DOI:** 10.17533/udea.iee.v39n1e05

**Published:** 2021-03-05

**Authors:** Isai Arturo Medina Fernández, Sonia Carreño Moreno, Lorena Chaparro Díaz, Ruth Magdalena Gallegos-Torres, Josué Arturo Medina Fernández, Eva Kerena Hernández Martínez

**Affiliations:** 1 Nurse, Masters. Professor. Universidad Autónoma de Coahuila, México. Email: isai-medina@uadec.edu.mx Universidad Autónoma de Coahuila Universidad Autónoma de Coahuila Mexico isai-medina@uadec.edu.mx; 2 Nurse, PhD in Nursing. Associate professor. Universidad Nacional de Colombia. Email: spcarrenom@unal.edu.co. Corresponding author Universidad Nacional de Colombia Universidad Nacional de Colombia Colombia spcarrenom@unal.edu.co; 3 Nurse, PhD in Nursing. Associate professor. Universidad Nacional de Colombia. Email: olchaparrod@unal.edu.co Universidad Nacional de Colombia Universidad Nacional de Colombia Colombia olchaparrod@unal.edu.co; 4 Nurse, PhD. Professor. Universidad Autónoma de Querétaro, México. Email: isisrmgx@gmail.com Universidad Autónoma de Querétaro Universidad Autónoma de Querétaro Mexico isisrmgx@gmail.com; 5 Nurse, Master. Professor. Centro Universitario Siglo XXI, México. Email: josuemedinafernandez@outlook.es Centro Universitario Siglo XXI Centro Universitario Siglo XXI Mexico josuemedinafernandez@outlook.es; 6 Nurse, PhD. Professor. Universidad Autónoma de Coahuila, México. Email: kerenahernandez@uadec.edu.mx Universidad Autónoma de Coahuila Universidad Autónoma de Coahuila Mexico kerenahernandez@uadec.edu.mx

**Keywords:** fear, stress, psychological, knowledge, students, nursing, nurses, coronavirus infections, pandemics, miedo, estrés psicológico, conocimiento, estudiantes de enfermería, enfermeras y enfermeros, infecciones por coronavirus, pandemias, medo, estresse psicológico, conhecimento, estudantes de enfermagem, enfermeiras e enfermeiros, infecções por coronavirus, pandemias

## Abstract

**Objective.:**

The study sought to correlate fear, stress, knowledge regarding COVID-19 in Nursing students and recent graduates in Mexico**.**

**Methods.:**

Correlational design, sample comprising 912 nursing students and graduates during the last 18 months from public and private universities of Mexico. To measure the variables, the study applied the instrument *Fear of COVID-19 Scale*, knowledge subscale of the scale *Knowledge, attitudes, and practices towards COVID-19*, and the instrument *COVID Stress Scale*.

**Results.:**

Relationship was found of the age variable with fear, danger of contamination, traumatic stress, knowledge and minor socioeconomic consequences (*p*<0.05). Likewise, relationship was observed of fear with stress regarding COVID-19, danger of contamination, socioeconomic consequences, xenophobia, traumatic stress, and compulsive checking (*p*<0.05). Stress and knowledge explain the presence of fear regarding COVID-19 in 50.3%, and fear and knowledge explain stress regarding COVID-19 in 50.4%.

**Conclusion.:**

Nursing students and recent graduates have high levels of stress and fear, besides low level of knowledge. The presence of high stress and low knowledge predict fear regarding COVID-19. Interventions are required on knowledge, stress, and fear regarding COVID-19 in the population studied.

## Resumen

**Objetivo.:**

Correlacionar el temor, estrés, conocimientos frente al COVID-19 en estudiantes y recién egresados de enfermería en México**.**

**Métodos.:**

Diseño correlacional, muestra constituida por 912 estudiantes de enfermería y egresados en los últimos 18 meses de universidades públicas y privadas de México. Para medir las variables se aplicó el instrumento *Fear of COVID-19 Scale*, subescala de conocimientos de la escala *Knowledge, attitudes, and practices towards COVID-19*, y el instrumento *COVID Stress Scale*.

**Resultados.:**

Se encontró relación de la variable edad con temor, peligro de contaminación, estrés traumático, conocimientos y menores consecuencias socioeconómicas (*p*<0.05). De igual forma, se observó relación del temor con estrés ante el COVID-19, peligro de contaminación, consecuencias socioeconómicas, xenofobia, estrés traumático, y comprobación compulsiva (*p*<0.05). El estrés y los conocimientos explican la presencia del temor ante el COVID-19 en un 50.3 %, así como el temor y conocimiento explican el estrés ante el COVID-19 en un 50.4 %.

**Conclusión.:**

Los estudiantes y recién egresados de enfermería tienen altos niveles de estrés y temor, además de bajo nivel de conocimientos. La presencia de alto estrés y bajos conocimientos predicen el temor ante del COVID-19. Se requieren intervenciones sobre los conocimientos, estrés y temor ante el covid-19 en la población estudiada.

## Resumo

**Objetivo.:**

Correlacionar medo, estresse, conhecimento contra COVID-19 em estudantes e recém-formados em enfermagem no México.

**Métodos.:**

Desenho correlacional, amostra composta por 912 estudantes e graduados de enfermagem nos últimos 18 meses de universidades públicas e privadas do México. Para mensurar as variáveis, foram aplicados o instrumento Escala de Medo do COVID-19, a subescala Conhecimento da escala Conhecimento, atitudes e práticas em relação ao COVID-19 e o instrumento Escala de Estresse COVID.

**Resultados.:**

Foi encontrada relação entre a variável idade e medo, perigo de contaminação, estresse traumático, conhecimento e menores consequências socioeconômicas (*p*<0.05). Da mesma forma, foi observada relação entre medo e estresse frente ao COVID-19, perigo de contaminação, consequências socioeconômicas, xenofobia, estresse traumático e verificação compulsiva (*p*<0.05). O estresse e o conhecimento explicam a presença do medo do COVID-19 em 50.3%, assim como o medo e o conhecimento explicam o estresse do COVID-19 em 50.4%.

**Conclusão.:**

Estudantes de enfermagem e recém-formados apresentam altos níveis de estresse e medo, bem como baixo nível de conhecimento sobre o COVID-19. A presença de alto estresse e baixo conhecimento prediz medo de COVID-19. Se faz necessário intervenções sobre o conhecimento, estresse e medo de covid-19 na população estudada.

## Introduction

The SARS-CoV-2 infection evolved into a pandemic of unforeseen dimensions due to its rate of spread, lack of knowledge of its mechanisms of infection and survival, besides the lack of global habits with respect to social distancing. In Mexico, the SARS-CoV-2 infection began with a case imported from Italy in February 2020, a situation upon which the Government of Mexico decreed the health emergency and its consequential measures of containment and mitigation of the spread of the virus. Since the confirmation of the first case and documentation of the first death due to COVID-19 in March 2020, the Mexican population in general began to fear upon this infection, developing behaviors of panic, such as excessive purchases and looting, which, together with the confinement order issued by the National Government, triggered different impacts, among them, affectation and fall of the economy.(1) However, fear regarding COVID-19 is not exclusive of the general population, given that it is a fact that one of the populations most vulnerable to the infection are health professionals, particularly those spending more time in the patient’s environment. According to this, Mexico has reported high affectation of health professionals due to COVID-19, with over 2000 infected by 09 May 2020. Added to the figures of infection through COVID-19, complaints are known from staff working in health institutions, which expose the weakened, disorganized, and saturated Mexican health system, as well as the lack of personal preparation to address the pandemic and the lack of access to personal protection elements.(2)

Given the novelty of SARS-CoV-2, the scant information the world still has about its spread, control, and treatment, health professionals in general have situations of fear and stress regarding COVID-19. Fear, as an unpleasant mental state produced by the perception of danger, has been documented in situations of pandemic; particularly in COVID-19, related suicide attempts have been documented.(3) However, in spite of the unpleasantness of the sensation, fear can be a protective factor, given that it moves humans toward prevention behaviors.(4) In turn, stress regarding COVID-19 has been reported in health workers, especially in physicians and nurses of first line of care. Severe stress conditions with repercussion in mental symptoms were reported in a sample of health workers in Europe, observing as associated factors prior mental symptoms and the proximity with infected patients.(5)

The principal causes of stress related with COVID-19 is the sense of danger, the possibility of self-inoculation of the virus, concern for the possibility of infecting relatives, and sleep alterations.(6) Specifically in Nursing, a study conducted in Wuhan, China reported high levels of stress and anxiety, upon which nurses reported as cause, that of having to leave their children alone and the high work load. Said study concluded about the importance of adding evidence in this area, seeking to develop better strategies of emotional containment and support.(7)

Given the critical situation regarding COVID-19, it is imperative to modify knowledge, attitudes, and practices in this context. In this respect, studies of this type have been carried out in health professionals, students and general population. In health professionals, lack of staff training is highlighted regarding the approach and prevention of COVID-19,(8) besides the need to increase knowledge, given that inadequate knowledge was reported by 10% of the professionals studied; attitudes of fear toward the virus, and the need to strengthen infection prevention measures.(9) With respect to students from health sciences, adequate knowledge was reported by 85%, prevention behaviors by 94%, and a perception of moderate risk.(10)

Together with the concerns and uncertainty of the disease, presence of anxiety and fear of contracting COVID-19, epidemiological change still not under control, nursing students have equal concerns on the ambiguity regarding these new roles that can limit the learning opportunities, given the need to consolidate the abilities and skills necessary to permit a transition.(11) According with the aforementioned, the need is observed to enhance evidence available on COVID-19 not only in terms of its genetics, replication, spread and control, but also in terms of matters related with the population confronting it and its consequences on mental health. The purpose of this study was to correlate fear, stress, knowledge with regards to COVID-19 in Nursing students and recent graduates in Mexico.

## Methods

This was a correlational, predictive, cross-sectional study conducted during May and June 2020 in Mexico. It used convenience sampling with 912 participants, which corresponded to undergraduate students (*n* = 711) and recent graduates from the Nursing program within a maximum period of 18 months (*n* = 201), being 18 years of age or more.

The study was carried out in the southeast, northeast, and central regions of Mexico. The characterization and measurement instruments were delivered via E-mail or shared through social media through Google forms; the average response time for the package of instruments was 30 minutes. The study was approved by the Bioethics Committee of the Faculty of Nursing at Universidad Autónoma de Coahuila with registry number CBFEUSUADEC-IEM2, respecting anonymity and confidentiality by applying the electronic informed consent.

The variables were analyzed with a participant characterization file and three instruments were applied. Permission was obtained from the authors to use the instruments. The instruments were translated into Spanish by an official translator and, thereafter, were adjusted in semantics by the authors to be used within the Mexican context: (i) *Fear of COVID-19 Scale,* this scale is made up of ten items, with one-dimensional behavior in the construct validity, which reported factor loads from 0.66 and above and an item-total correlation > 0.4. In its original version, it reports a Cronbach’s alpha of 0.82 and an interclass correlation coefficient of 0.72, which is adequate for its stability. It has a five-point Likert-type response scale, from never to always. The scale was designed to be self or hetero administered and the results indicated that a higher score means greater fear regarding COVID-19;(12) (ii) *COVID Stress Scale*, which measures five dimensions established through confirmatory factor analysis; these being danger regarding COVID-19 and contamination, economic consequences, xenophobia, traumatic stress, and compulsive behavior. Cronbach’s Alpha internal consistency was > 0.8 in total and in all dimensions,(13) where a higher score meant greater stress regarding COVID-19; and (iii) *Knowledge, attitudes, and practices towards COVID-19*, which is constituted by 12 questions of which four refer to the clinical aspects of the disease, three are about the transmission mechanisms, and five are about prevention and control mechanisms. The questions have response options of false, true, and don’t know. Likewise, the score of the scale goes up to 12 points, where each question has a value of one point, the result indicates that a higher score means greater knowledge. In its original version, the scale has a Cronbach’s alpha of 0.71,(14) however, this study only applied the knowledge subscale.

Moreover, the description of the sample and analysis of the central variables of the study used absolute and relative frequencies for the discrete variables, besides central tendency measurements, dispersion and 95% confidence intervals for continuous variables. Correlations were explored among the continuous variables from Pearson’s statistics, with prior compliance of normality requirements. Additionally, the Mann Whitney U was used to identify difference of measurements of stress, fear, and knowledge between groups. The variables included in the regression models were those reporting correlations and those the literature showed as relevant in the analysis.

## Results

The study obtained 956 surveys from subjects of which 44 were discarded because they were under age. The sample showed that 78% (*n* = 711) of the participants were currently studying in the Nursing career and 22% (*n* = 201) had recently graduated, who were in the social service or had already finished (15.2%) or were within a year from having concluded their studies without having received their title (6.8%). Of all the participants, 33.7% live in Yucatán (southern Mexico), 32.1% of the students live in the state of Coahuila (northern Mexico), 15.2% live in Zacatecas (central Mexico), and the rest is distributed in the rest of the states of the Mexican Republic.

A total of 77.7% of the participants were women and 22.3% were men. The mean age was 21 ±2.25 years. Regarding involvement with COVID-19, only 4.7% of the participants were currently working in an area that cares for these patients and 66.1% of the subjects has taken some course related with prevention, diagnosis and/or treatment of the pathology.

Notably, 92.4% of the students indicated not having manifested symptoms, like coughing, sore throat, fever, or general discomfort in the last 14 days; 14.8% had been in contact with someone classified as suspected of having the disease and 6.6% with someone identified as positive case. With respect to whether the students live with someone considered at risk, it is highlighted that 57.6% indicated living with someone enduring a chronic disease and 35.6% with an elderly adult; 15.4% of the participants reported living with someone who Works directly in a health institution.

In addition, Table 1 identifies the descriptive statistics of the variables, with high scores for fear, stress, and knowledge regarding COVID-19.

**Table 1:** Descriptive statistics of fear, stress and knowledge regarding COVID-19

Scale / subscales	M	SD	Min	Max	95%CI of the median	95%CI of the median
Scale / subscales	M	SD	Min	Max	LL	UL
Fear regarding COVID-19	25.71	6.90	11	50	25.26	26.16
Stress regarding COVID-19	98.22	25.47	37	180	95.57	98.88
Danger of contamination	39.34	10.01	12	60	38.69	39.99
Socioeconomic consequences	18.15	6.24	6	30	17.75	18.56
Xenophobia	17.15	6.35	6	30	16.73	17.56
Traumatic stress	10.68	5.12	6	30	10.35	11.01
Compulsive checking	12.89	4.81	6	30	12.58	13.20
Knowledge regarding COVID-19	8.24	1.04	3	12	8.17	8.31

Note: M = median, SD = Standard deviation, Min = minimum value, Max = maximum value, 95%CI = Confidence interval at 95%, LL = lower limit, UL = upper limit.

It was found that fear and stress are different according to sex, being higher in women (*p* <0.05); with respect to being a student or recent graduate, stress and knowledge were significantly different (*p* <0.001), with stress being higher in students and recent graduates having greater knowledge. Likewise, it was found that being in contact with a case suspected of COVID-19 has greater knowledge with respect to those who have not has said contact (*p* <0.05). Similarly, the group of individuals who have taken a course related with COVID-19 has less fear and stress, as well as greater knowledge about the disease (*p* <0.05).

Table 2 shows the relationships among age, fear, stress, and knowledge regarding COVID-19 and their subscales. Furthermore, it was found that stress and knowledge are predictors of fear regarding COVID-19, as well as fear and knowledge about stress regarding COVID -19 and stress, fear, having been in contact with someone, as well as having taken some course related with the disease are predictors for knowledge regarding COVID-19 (Table 3). Two models contribute to the explained variance of stress and fear regarding COVID-19 with over 50%, and knowledge regarding COVID-19 was explained by 5.1%.

**Table 2:** Correlation of fear, stress, and knowledge regarding COVID-19

Variables	1	2	3	4	5	6	7	8	9
1- Age	1								
2- Fear regarding COVID-19	0.096**	1							
3- Stress regarding COVID-19	0.036	0.684**	1						
4- Danger of contamination	0.101*	0.657**	0.880**	1					
5-Socioeconomic consequences	-0.074*	0.421**	0.780**	0.582**	1				
6- Xenophobia	-0.031	0.652**	0.767**	0.680**	0.595**	1			
7- Traumatic stress	0.074**	0.450**	0.650**	0.503**	0.373**	0.331**	1		
8- Compulsive checking	-0.043	0.450**	0.625**	0.429**	0.394**	0.302**	0.501**	1	
9- Knowledge regarding COVID-19	0.070*	0.019	-0.055	-0.036	-0.085*	-0.080*	-0.003	0.044	1

(*) *p* <0.05, (**) *p* <0.001

**Table 3:** Model of prediction factors of stress, fear, and knowledge regarding COVID-19

	Fear	Fear	Fear	Fear	Fear	Stress	Stress	Stress	Stress	Stress	Knowledge	Knowledge	Knowledge	Knowledge	Knowledge
	*R* ^*2*^	*R* ^*2*^	*F*	*F*	*p*	*R* ^*2*^	*R* ^*2*^	*F*	*F*	*p*	*R* ^*2*^	*R* ^*2*^	*F*	*F*	*p*
	0.503	0.503	130.89	130.89	<0.001	0.504	0.504	131.24	131.24	<0.001	0.051	0.051	6.89	6.89	<0.001
	*B*	TE	*β*	*t*	*p*	*B*	TE	*β*	*t*	*P*	*B*	TE	*β*	*T*	*p*
Fear regarding COVID-19						2.60	0.087	0.705	29.92	0.001	0.017	0.007	0.111	2.41	0.016
Stress regarding COVID-19	0.191	0.006	0.706	29.92	0.001						-0.004	0.002	0.091	-1.984	0.048
Knowledge regarding COVID-19	0.384	0.159	0.058	2.41	0.016	-1.16	0.586	-0.048	-1.98	0.048					
Having symptoms of COVID-19 in the last 14 days	0.336	0.623	0.013	0.539	0.590	-0.703	2.29	-0.007	-0.306	0.760	-0.108	0.130	-0.027	-0.829	0.407
Having contact with someone suspected of COVID-19	0.769	0.554	0.040	1.38	0.165	-2.78	2.40	-0.039	-1.36	0.178	0.389	0.115	0.132	3.379	0.001
Having contact with someone diagnosed with COVID-19	-0.601	0.785	-0.022	-0.766	0.444	2.19	2.89	0.021	0.758	0.448	-0.145	0.164	-0.035	-0.888	0.375
Relative health worker	-0.509	0.452	-0.027	-1.12	0.260	2.02	1.66	0.029	1.21	0.224	-0.027	0.094	-0.009	-0.290	0.772
Having taken a course on COVID-19	-0.319	0.350	-0.022	-0.912	0.362	-2.06	1.28	-0.038	-1.60	0.109	0.375	0.072	0.170	5.200	0.000

*Note*: TE: Typical error; *β:* beta; *F*= Snedecor’s F, *t* = Student’s t, *p* = significance level

## Discussion

The aim of this study was to correlate fear, stress, and knowledge regarding COVID-19 in Nursing students and recent graduates in Mexico. Nursing students in training are confronting diverse educational challenges caused by interrupted studies and modified learning strategies, besides going through situations related with the pandemic that could affect their mental health due to the presence of stress, fear, and poor knowledge about COVID-19. With respect to fear regarding COVID-19, this study obtained a median of 25.7; said result was higher than that reported by Winter *et al*.,(15) and Sakib *et al*.,(16) with a median of 2.75 and M = 18.3, respectively. That fear was greater in contrast with the literature; it could have been generated by uncertainty for students with anguish due to social isolation, anguish due to fear of contracting the disease, and moral anguish as a consequence of seeing death at large scale.(11) However, although the nursing students have knowledge about the disease, fear may be related with the uncertainty of future close contact with patients, lack of knowledge of real settings, and discomfort caused by special protection, upon the suffering and death of critical patients and fear of infecting the family.(17) Stress regarding COVID-19 obtained a median of 98.2, which is higher than that reported by Asmundson *et al.,(*18) with a median of 41.7 and 67.7 in the study by Lakaran *et al.*(19) Presence of a higher median may be related with the sense of security of people in each country, as well as the uncertainty of not having answers about the end of the pandemic, constant exposure to a flow of information on COVID-19 and its effects, and its dissemination in social relations and constant repetition of recommendations and prohibitions, all of which can affect negatively the mental health of the individuals.(18)

Regarding knowledge of COVID-19, the median in this study was 8.24, which is lower than that reported by Zhong *et al.*,(14) Azlan with a median of 10.5(20), and Begum(21) with a median of 10.8. The median was below that of various Asian countries; this indicates the possibility that students do not have appropriate practices to prevent COVID-19, which suggests that programs on health education must improve knowledge on the disease to help to promote an optimistic attitude and maintain safe practices.(22) With respect to the relation of knowledge and stress in our study, no relation was found; this was represented to the contrary by Maarefvand *et al*.,(23) where stress levels were related with preventive knowledge for COVID-19. Likewise, Zhi *et al.,*(24) suggested that stress levels are inversely proportional to knowledge in effective ways of managing the pandemic.

Our study found no relation between fear and knowledge on COVID-19, which was similar to the study by Ali *et al.*(25) In contrast, Hossain *et al.,*(26) reported that with greater knowledge, fear regarding COVID-19 is lower, which could support the hypothesis that lack of knowledge on a specific area could facilitate the construction or ingrained beliefs of myths of such. Thus, knowledge on COVID-19 is a prior requisite to establish prevention beliefs, form positive attitudes, and promote positive behaviors and cognition and the attitudes of people toward the disease affect the effectiveness of their coping strategies and behaviors to a certain extent.(27) Together with the aforementioned, this study found that fear is a predictor of stress regarding COVID-19; these results were similar to those by Bakioğlu(28) and Begum *et al*.(29) This may explain that fear is a strong emotion that affects physical responses, cognitive abilities, and mood of individuals, generating greater concern and further worsening the severity of the situation lived by the person, besides the impact on the mental health of Nursing students and professionals could be severe, given that they experience constant psychological affectation, caused by their work in the first line or worry for an uncertain future in the work environment.

In conclusion, higher scores are noted in the sample in this study than those reported by studies in other countries for fear and stress regarding COVID-19; as well as lower level of knowledge. Low knowledge and high stress predict high levels of fear regarding COVID-19. Likewise, the high percentage of influence over the variables indicates the need for up-to-date interventions on the pathology and follow up and care of the mental health of students and recent graduates in Mexico.
